# Tributyrin Plays an Important Role in Regulating the Growth and Health Status of Juvenile Blunt Snout Bream (*Megalobrama amblycephala*), as Evidenced by Pathological Examination

**DOI:** 10.3389/fimmu.2021.652294

**Published:** 2021-04-12

**Authors:** Hualiang Liang, Ke Ji, Xianping Ge, Bingwen Xi, Mingchun Ren, Lu Zhang, Xiaoru Chen

**Affiliations:** ^1^ Key Laboratory for Genetic Breeding of Aquatic Animals and Aquaculture Biology, Freshwater Fisheries Research Center (FFRC), Chinese Academy of Fishery Sciences (CAFS), Wuxi, China; ^2^ Wuxi Fisheries College, Nanjing Agricultural University, Wuxi, China; ^3^ Tongwei Co., Ltd., Chengdu, China; ^4^ Healthy Aquaculture Key Laboratory of Sichuan Province, Chengdu, China

**Keywords:** juvenile blunt snout bream, tributyrin, growth, antioxidant capacity, immune responses

## Abstract

The present study aimed to assess the role of tributyrin (TB) in regulating the growth and health status of juvenile blunt snout bream (*Megalobrama amblycephala*) through an 8-week feeding experiment. Six groups were fed experimental diets with added TB percentages of 0% (control group), 0.03%, 0.06%, 0.09%, 0.12% and 0.15%. The present results showed that TB supplementation in feed had some positive impacts on FW, WG, FCR and SGR, and the best results were found in the 0.06% TB group (*P<0.05*). However, TB supplementation in feed had no significant effects on SR, CF, VSI or whole-body composition (*P>0.05*). TB supplementation in feed increased antioxidant capacity and immunological capacity and attenuated the inflammatory response by increasing the activity of T-SOD, GPx, CAT and the levels of anti-inflammatory cytokines (IL-10 and TGF-β) and decreasing the levels of MDA and anti-inflammatory cytokines (TNF-α) (*P<0.05*). Furthermore, TB supplementation improved immunity by increasing the levels of immunoglobulins (IgM and IgG), C3 and IFN-γ (*P<0.05*). Surprisingly, 0.06%-0.12% TB supplementation significantly increased the content of IL-1β (*P<0.05*). However, TB supplementation in feed had no significant effects on the plasma content of GSH, HSP70, IL-8 and the activity of T-AOC (*P>0.05*). The possible mechanism was that TB activated PI3K/Akt/Nrf2 and inhibits the NF-κB signaling pathway, further regulating the mRNA levels of key genes with antioxidant capacity and the inflammatory response; for example, it increased the mRNA levels of Nrf2, Cu/Zn-SOD, HO-1, CAT, Akt, PI3K, GPx, IL-10, and TGF-β and decreased the mRNA levels of NF-κB and TNF-α (*P<0.05*). In addition, 0.06%-0.15% TB supplementation significantly increased the mRNA levels of IL-1β (*P<0.05*). TB supplementation in feed had no significant effects on the mRNA levels of HSP70, Mn-SOD and IL-8 (*P>0.05*). Evidence was presented that TB supplementation decreased the mortality rate caused by *Aeromonas hydrophila* challenge. In pathological examination, TB supplementation prevented hepatic and intestinal damage. Generally, TB supplementation improved the growth performance of juvenile blunt snout bream. Furthermore, TB supplementation activated PI3K/Akt/Nrf2 and inhibited the NF-κB signaling pathway, regulating health status and preventing hepatic and intestinal damage.

## Introduction

Butyric acid is a short-chain fatty acid in the intestinal tract and an important energy source of the colon (1). Butyric acid plays important roles in regulating growth performance, gastrointestinal function, immunity, gastrointestinal microecological balance and intestinal pH, and it has bactericidal and bacteriostatic functions (2, 3). Despite its many functions, the direct use of butyric acid as a liquid is very limited because of its volatility, corrosivity, very unpleasant odor and excessive absorption rate before its arrival in the small intestine. These disadvantages have greatly limited the popularization and application of butyric acid in farmed animal feed. To solve this industry problem, butyric acid is mostly added in the form of sodium butyrate; however, this practice does not solve many problems, such as easy delirium and unpleasant odor of butyric acid (4, 5). Hence, applications remain limited in husbandry and aquaculture. In recent years, tributyrin (TB), the precursor of butyric acid and a colorless oily liquid, has been found to address the unfavorable characteristics of butyric acid very well and has better palatability in that it has almost no smell or a slightly fatty fragrance; thus, it has great application potential in aquaculture (6–8).

TB is composed of trimolecular butyric acid and a molecule of glycerol, which is a short-chain fatty acid ester. Under the action of intestinal lipase, it is decomposed into butyric acid, glycerol butyrate and oil. Hence, TB has a function similar to that of butyric acid. Some studies have shown that TB has a positive effect on growth performance in various terrestrial animals, such as broiler chickens (1, 9), weaned pigs (10, 11), and small tail sheep ewes (12). In addition, TB can promote the healthy development, digestion and collection of nutrients of the intestinal tract. TB supplementation can improve the development of the intestine, including by increasing villus height, villus width, muscular thickness and villus area in the duodenum, while significantly decreasing crypt depth in intrauterine growth‐retarded piglets (13). It has been found to have similar results in nursery pigs (14). The improvements in intestinal development may be closely related to the immune state of the intestine. Studies have shown that the breakdown of TB into butyric acid can promote hydrogen ion accumulation and reduce intestinal pH, which can destroy the membranes of some harmful bacteria and stimulate the breeding of beneficial acidophilic microorganisms and the development of the intestinal mucosa (15). Furthermore, TB can improve intestinal immunity by regulating the antioxidant system and the inflammatory factor system (16, 17). There are only a few reports that TB supplementation in feed also has positive effects on growth performance, intestinal development and immunity in aquatic animals, such as snakehead (*Channa argus*) (18), black sea bream (*Acanthopagrus schlegelii*) (19) and yellow drum (*Nibea albiflora*) (20, 21). However, research on the effects of TB mainly focuses on livestock and poultry, and aquatic animals are rarely reported, and the mechanisms of growth and immune regulation are still unclear in aquatic animals.

As an important economic fish in China, blunt snout bream (*Megalobrama amblycephala*) has many advantages such as delicious meat, high economic value, and fast growth; thus, it is widely distributed all over the world and loved by many consumers. However, with the expansion of farming scale, diseases have begun to break out, resulting in increased mortality and economic losses, especially in summer (22). Therefore, to improve growth and health status, studies on the anti-stress function of feed are urgently needed. To date, no studies regarding the effects of TB supplementation on blunt snout bream have been reported, and the mechanism by which TB regulates health status is also unclear. Hence, the present study investigated the effect of TB supplementation on growth performance, plasma antioxidant and immune capacity indexes, PI3K/Akt/Nrf2 signaling and inhibition of the NF-κB signaling pathway and performed pathological examinations to assess the role of TB in regulating health status and to elucidate the related mechanism in juvenile blunt snout bream.

## Materials and Methods

### Experimental Ethics Statement

The protocols used on the experimental fish followed the guidelines of the Institutional Animal Care and Ethics Committee of Nanjing Agricultural University, Nanjing, China. [Permit number: SYXK (Su) 2011-0036].

### Diets

Commercial feed was used in the experiment. TB (50% active ingredient) was purchased from Qingdao Keneng Biotechnology Co., Ltd. (Qingdao, China). Six groups were fed experimental feed with different percentages of TB: 0% (the control group), 0.03%, 0.06%, 0.09%, 0.12% and 0.15%. The main protein sources and lipid sources were listed in **Table 1**. The ingredients were crushed, proportioned according to the feed formula, mixed and then granulated though a granulator (F-26 (II), South China University of Technology, Guangzhou, China). After granulation, the feed was dried in a drying oven at 65°C.

**Table 1:** Composition of the basal diet (% dry matter).

Basal Ingredients			
Fish meal^a^	4	Soybean oil	5.5
Soybean meal^a^	23	Monocalcium phosphate	1.8
Rapeseed meal^a^	24	Vitamin and mineral premix^b^	1.5
Cottonseed meal^a^	9	Vitamin C (35%)	0.1
Wheat meal	20	Choline chloride (65%)	0.1
Rice bran	11		
Composition of feed (dry matter)			
Protein (%)	33.15		
Lipid (%)	8.78		
Energy (KJ/g)	17.89		

a Fish meal, Rapeseed meal, Soybean meal, Cottonseed meal, Wheat meal, Rice meal obtained from Wuxi Tongwei feedstuffs Co., Ltd. (Wuxi, China).

b Main vitamin and mineral mix of vitamin and mineral premix (IU or mg/ kg of diet): ①The main component of vitamin in premix: Vitamin A, 900 000 IU; Vitamin D, 250 000 IU; Vitamin E, 4500 mg; Vitamin K 3 , 220 mg; VitaminB 1, 320 mg; Vitamin B 2 , 1090 mg; Vitamin B 5 , 2000 mg; Vitamin B 6, 5000 mg; Vitamin B 12 , 116 mg; Pantothenate, 1000 mg; Folic acid,165 mg; Choline, 60 000 mg; Biotin, 50 mg; Niacin acid, 2500 mg. ②The main mineral component in premix: calcium phosphate, 20 g; sodiumchloride, 2.6 g; potassium chloride, 5 g; magnesium sulphate, 2 g; ferrous sulphate, 0.9 g; zinc sulphate, 0.06 g; cupric sulphate, 0.02 g; manganese sulphate, 0.03 g; sodium selenate, 0.02 g; cobalt chloride, 0.05 g; potassium iodide, 0.004g. Zeolite was used as a carrier in premix.

### Experimental Procedure

Juvenile blunt snout bream based on health (lively, no scars, no signs of bleeding) and similar size were obtained from the breeding base of the Freshwater Fisheries Research Center (FFRC) and acclimatized to the experimental conditions in recirculatory tanks (φ820×700mm) for two weeks. The juvenile fish (6.51 ± 0.01 g) were randomly distributed into 18 recirculatory tanks (in triplicate). During the period of breeding, the fish were fed three times a day (at 08:00, 12:00 and 16:00) until apparent satiation by visual observation of the feeding behavior of the fish. The water quality was measured once a week with an YSI ProPlus multiparameter water quality analyzer (YSI China, Hong Kong, China). The water temperature was kept at 28.45 ± 0.41°C, the pH at 7.31 ± 0.16, the ammonia nitrogen concentration at 0.027 ± 0.003 mg/L, and the dissolved oxygen concentration at 6.13 ± 0.40 mg/L. The light exposure was controlled with a photoperiod of 12L:12 D (light: dark).

### Sample Collection

After a 56-day feeding trial, the fish were fasted for 24 h to allow evacuation of the contents of the alimentary tract. Two fish (per recirculation tank) were obtained for whole-body analysis. Furthermore, three fish (per recirculation tank) were randomly selected and anesthetized using 100 mg/L MS-222. Immediately, blood samples were collected from the caudal vein using an injection syringe and centrifuged at 3,500 x g at 4°C for 10 minutes for collection of plasma. The intestine was also collected for gene expression analysis and pathological analysis, and the liver was collected for pathological analysis (the detailed collection methods are shown in section 2.7 on hematoxylin and eosin (HE) staining). The collected intestine samples were stored at -80°C until analysis.

### Proximate Composition and Plasma Biochemical Analysis

Based on the established methods of the AOAC (23), the chemical compositions of the dried whole fish, ingredients and experimental diets were assessed with regard to lipids (ID 991.36), crude protein (ID 984.13), ash (ID 923.03) and moisture (ID 920.36). Enzyme-linked immunosorbent assays (ELISAs) were used for evaluation of interferon-γ (IFN-γ), heat stress protein 70 (HSP70), immunoglobulin M (IgM), immunoglobulin G (IgG), complement component 3 (C3), interleukin 10 (IL-10), transforming growth factor-β (TGF-β), tumor necrosis factor-α (TNF-α), interleukin 1β (IL-1β) and interleukin 8 (IL-8) in plasma. The methods, kits and equipment used in this study were presented in **Table 2**.

**Table 2:** The chemical analysis used in the experiment.

Items	Methods	Assay Kits/Testing equipment
*Composition of diets/ingredients*		
Moisture	Oven method	Electric blast drying oven (Shanghai Yiheng Scientific Instrument Co., Ltd., Shanghai, China)
Protein	Kjeldahl	Auto kieldahl apparatus: Hanon K1100 (Jinan Hanon Instruments Co., Ltd., Jinan, China)
Lipid	Soxhlet	Auto fat analy: Hanon SOX606 (Jinan Hanon Instruments Co., Ltd., Jinan, China)
Gross energy	Combustion	Oxygen bomb calorimeter: IKA C6000 (IKA WORKS GUANGZHOU, Guangzhou, China)
*Plasma parameters related antioxidant capacity*		
T-SOD^1^	WST-1 method	Assay kits purchased from Jian Cheng Bioengineering Institute (Nanjing, China); Spectrophotometer (Thermo Fisher Multiskan GO, Shanghai, China).
T-AOC^2^	ABTS method	
GSH^3^	Microplate method	
GPx^4^	Colorimetric method	
MDA^5^	TBA method	
CAT^6^	Ammonium molybdenum acid method	
*Plasma parameters related ELISA*		
IL-10^7^	Double antibody sandwich method (Test wavelength 450nm)	Assay kits purchased from Jiangsu Enzyme-Free Industrial Co. LTD (Yancheng, China); Thermo Scientific Microplate Reader-1510 (Thermo Fisher Scientific, Shanghai, China).
TGF-β^8^		
IFN-γ^9^		
HSP70^10^		
IgM^11^		
IgG^12^		
C3^13^		
TNF-α^14^		
IL-1β^15^		
IL-8^16^		

^1^T-SOD, total superoxide dismutase; ^2^T-AOC, total antioxidant capacity; ^3^GSH, glutathione; ^4^GPx, glutathione peroxidase; ^5^MDA, malondialdehyde; ^6^CAT, catalase; ^7^IL-10, interleukin 10; ^8^TGF-β, transforming growth factor-β; ^9^IFN-γ, interferon-γ; ^10^HSP70, heat stress protein 70; ^11^IgM, immunoglobulin M; ^12^IgG, immunoglobulin G; ^13^C3, complement component 3; ^14^TNF-α, tumor necrosis factor-α; ^15^IL-1β, interleukin 1β; ^16^IL-8, interleukin 8.

### Tissue RNA Extraction and Real-Time PCR Analysis

The method was described in our previous study (24). Tissue RNA was first extracted, and then the quality and quantity were determined. Later, the mRNA levels of all genes were tested with a 7500 real-time PCR machine (Applied Biosystems, USA). Initially, cDNA (2.0 μL) was reacted with 10.0 μL of SYBR^®^ Premix Ex Taq II (2×), 0.8 μL of forward and reverse primers (10 μM each), 0.4 μL of ROX reference dye II (50×), and 6.0 μL of RNase-free distilled water in a 20 μL final reaction volume. The primer sequences used for qRT-PCR were described in our previous study (25), and the specific primers for the target genes used are shown in **Table 3**. β-actin was used as a nonregulated reference gene, and no obvious change was observed in its gene expression (24, 26, 27). Relative gene expression was calculated using Pfaffl’s mathematical model for CT calculation (28).

**Table 3:** Primer sequence for qRT-PCR.

Target genes	Forward (5’-3’)	Reverse (5’-3’)	References
β-actin	TCGTCCACCGCAAATGCTTCTA	CCGTCACCTTCACCGTTCCAGT	Ji et al. (25)
PI3K^1^	AAGAAAGTTTGCCACACCGC	TTGTCCATGGTTCAGTGGCA	Ji et al. (25)
Akt^2^	GCTGGGTAAAGGCACGTTTG	CTCTCGGTGACCGTATGAGC	Ji et al. (25)
NF-κB^3^	AGTCCGATCCATCCGCACTA	ACTGGAGCCGGTCATTTCAG	Ji et al. (25)
Nrf2^4^	GGGGAAGTCCTTGAACGGAG	AACCAGCGGGAATATCTCGG	Ji et al. (25)
Keap1^5^	AATATCCGCCGGCTGTGTAG	TGAGTCCGAGGTGTTTCGTG	Ji et al. (25)
CAT^6^	CAGTGCTCCTGATACCCAGC	TTCTGACACAGACGCTCTCG	Ji et al. (25)
Cu/Zn-SOD^7^	AGTTGCCATGTGCACTTTTCT	AGGTGCTAGTCGAGTGTTAGG	Ji et al. (25)
HO-1^8^	TCACACCGGGAAACGAGAAG	TGGAGCATTTCTACGGCCAG	Ji et al. (25)
HSP70^9^	CGACGCCAACGGAATCCTAAAT	CTTTGCTCAGTCTGCCCTTGT	Ji et al. (25)
Mn-SOD^10^	AGCTGCACCACAGCAAGCAC	TCCTCCACCATTCGGTGACA	Ji et al. (25)
GPx^11^	GAACGCCCACCCTCTGTTTG	CGATGTCATTCCGGTTCACG	Ji et al. (25)
TNF-α^12^	TGGAGAGTGAACCAGGACCA	AGAGACCTGGCTGTAGACGA	Ji et al. (25)
IL-8^13^	CAGAGAGTCGACGCATTGGT	ATTCACGGTGCTTTGTTGGC	Ji et al. (25)
IL-1β^14^	TTCTTCCCCTCACCTGGTCT	CCAGCGCGAAGTTTGTCAAT	Ji et al. (25)

^1^PI3K, phosphoinositide 3-kinase; ^2^Akt, protein kinase B; ^3^NF-κB, nuclear factor-kappa B; ^4^Nrf2, nuclear factor erythroid 2-related factor 2; ^5^Keap1, Kelch-like ECH-associated protein 1; ^6^CAT, catalase; ^7^Cu/Zn-SOD, copper and zinc superoxide dismutase; ^8^HO-1, heme oxygenase 1; ^9^HSP70, heat stress protein 70; ^10^Mn-SOD, manganese superoxide dismutase; ^11^GPx, glutathione peroxidase; ^12^TNF-α, tumor necrosis factor-α; ^13^IL-8, interleukin 8; ^14^IL-1β, interleukin 1β.

### Hematoxylin and Eosin (HE) Staining

HE staining of liver and intestine samples was performed as described in our previous study (29). In brief, the method included (1) tissue fixation by 4% paraformaldehyde, (2) gradient alcohol dehydration and methyl salicylate clearing, (3) paraffin embedding, (4) slicing and patching, (5) HE staining, (6) dehydration sealing, and other procedures. Finally, pathological changes in the liver and intestine were observed and analyzed with a Zeiss microscope (Axioplan 2, Oberkochen, Germany).

### *Aeromonas hydrophila* Challenge Test

The remaining 15 fish from each tank were challenged with *Aeromonas hydrophila* (*A. hydrophila*). The method was described in our previous study (30). The pre-experiment was carried out before the challenged experiment. The five concentration gradients were set as 1x10^5^ CFU/mL, 1x10^6^ CFU/mL, 1x10^7^ CFU/mL, 1x10^8^ CFU/mL, 1x10^9^ CFU/mL, respectively. The half lethal concentration was determined to be 1x10^7^ CFU/mL. Whereafter, the *A. hydrophila* was resuspended and amplified by inoculation in nutrient broth and incubation in a shaker at 160 rpm (37°C) for 24 h. The challenge concentration w as adjusted to 1x10^7^ CFU/mL using a bacterial turbidimeter (SGZ-6AXJ, Yue Feng Instrument Co. Ltd., Shanghai, China). The fish were challenged by intraperitoneal injection with 1 mL/100 g (1% of body weight).

### Calculations and Statistical Analysis

Parameters were calculated based on the following equations:

Survival rate(SR, %)=100 ×(survival fish number/total fish number)

Weight Gain (WG, %)=100×[final weight (g)−initial weight (g)]/initial weight (g)

Specific grow rate (SGR, %/day)=100×[{Ln (final body weight (g)}−Ln {initial body weight (g)}/days]

Feed conversion ratio (FCR)=dry feed fed (g)/wet weight ain (g)

$$\text{Condition Factor }\left(\text{CF, g}/{\text{cm}}^{3}\right)=100\times \text{body weight }(\text{g})/\text{body length }{\left(\text{cm}\right)}^{3};$$

Visceral somatic index (VSI, %)=100 × visceral weight (g)/body weight (g);

The data were subjected to normality and homogeneity tests where necessary. Statistical analysis was performed using IBM SPSS Statistics 23. Tukey’s test was used to determine whether significant differences existed between means. The data are expressed as the mean with S.E (M± S.E).

## Results

### Growth Performance, Whole-Body Composition and Physique Parameters

TB supplementation in feed had some positive impacts on final weight (FW), weight gain rate (WG), feed conversion ratio (FCR) and specific growth rate (SGR), and the best results were found in the 0.06% TB group (*P<0.05*). However, TB supplementation in feed had no significant effect on survival rate (SR), condition factor (CF), visceral somatic index (VSI) or the whole-body compositions of protein, lipids, moisture and ash (*P>0.05*) (**Tables 4** and **5**).

**Table 4:** Effect of TB supplementation on growth performance and physical indexes of juvenile blunt snout bream^a^.

Addition level (%)	IW (g)	FW (g)	FCR^b^	WG (%)^c^	SGR (%/d)^d^	SR (%)^e^	CF (g/cm^3^)^f^	VSI (%)^g^
0	6.53±0.01	15.51±0.44^a^	1.49±0.03^b^	137.64±6.62^a^	1.37±0.04^a^	100±0.00	2.18±0.07	8.83±0.44
0.03	6.50±0.01	17.84±0.33^b^	1.32±0.02^a^	174.44±5.07^b^	1.60±0.03^b^	100±0.00	2.21±0.05	8.98±0.34
0.06	6.51±0.01	18.27±0.55^b^	1.31±0.01^a^	180.58±8.26^b^	1.61±0.02^b^	100±0.00	2.06±0.07	9.54±0.39
0.09	6.52±0.01	16.70±0.24^a^ ^b^	1.40±0.03^a^ ^b^	155.99±3.79^a^ ^b^	1.49±0.02^a^ ^b^	100±0.00	2.03±0.03	8.83±0.22
0.12	6.53±0.02	16.70±0.20^a^ ^b^	1.44±0.02^a^ ^b^	155.83±2.80^a^ ^b^	1.46±0.01^a^ ^b^	100±0.00	2.06±0.04	8.22±0.55
0.15	6.52±0.01	16.28±0.84^a^ ^b^	1.48±0.04^b^	149.80±12.98^a^ ^b^	1.42±0.06^a^	100±0.00	2.19±0.06	8.53±0.27

a All data are means of triplicate, value in the same column with different superscripts are significantly different (P ˂ 0.05).

b Feed conversion ratio (FCR) = dry feed fed (g)/ wet weight gain (g)

c Weight gain (WG) (%) = 100 × [final weight (g) -initial weight (g)] /initial weight (g)

d Specific growth rate (SGR) (%/d) =100×{ [Ln (final body weight (g)) –Ln (initial body weight (g) ) ]/days }

e Survival rate (SR) (%) =100× (survival fish number/total fish number)

f Condition factor (CF, g/cm^3^) = 100 ×body weight (g) / body length (cm)^3^;

g Visceral somatic index (VSI, %) = 100 × visceral weight (g) / body weight (g);

**Table 5:** Effect of TB supplementation on whole body composition of juvenile blunt snout bream^a^.

Addition level (%)	Moisture (%)	Protein (%)	Lipid (%)	Ash (%)
0	71.99±0.70	15.86±0.41	9.11±0.42	3.13±0.03
0.03	70.53±0.94	15.85±0.20	10.26±0.75	3.32±0.04
0.06	71.15±0.37	15.84±0.45	9.65±0.37	3.27±0.09
0.09	69.83±0.68	16.50±0.23	10.74±0.68	3.12±0.11
0.12	70.03±0.64	16.61±0.36	11.17±0.91	3.25±0.17
0.15	71.23±0.29	16.59±0.21	9.68±0.60	3.50±0.15

a All data are means of triplicate, value in the same column with different superscripts are significantly different (P ˂ 0.05).

### Plasma Biochemical Composition

**Figure 1:**
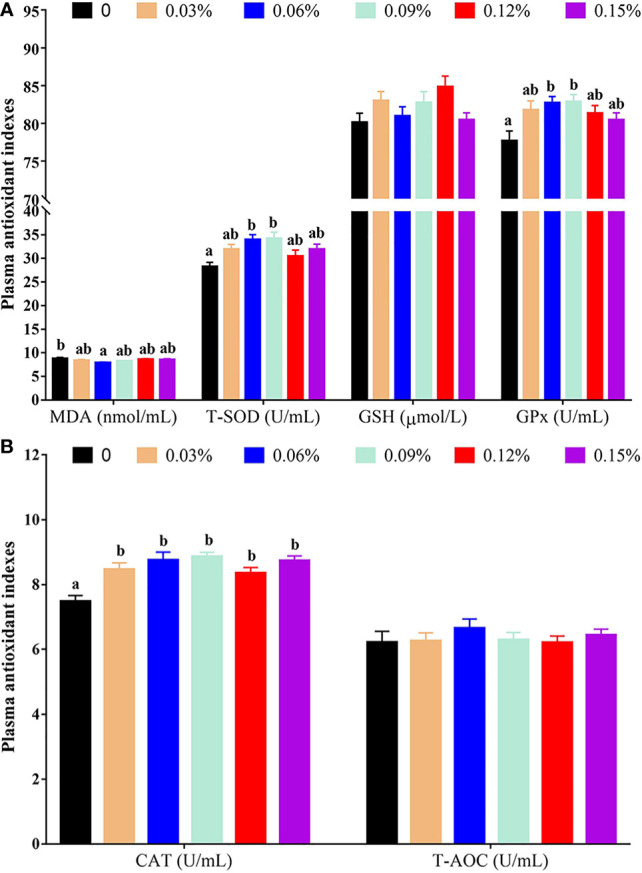
The results of plasma antioxidant indexes with different levels of TB supplementation. **(A)** MDA, malondialdehyde; T-SOD, total superoxide dismutase; GSH, glutathione; GPx, glutathione peroxidase; **(B)** CAT, catalase; T-AOC, total antioxidant capacity. Data are expressed as means with S.E. Value with different superscripts are significantly different (P < 0.05).

Compared with the control group (without TB supplementation, 0%), the group with 0.06% TB supplementation in feed exhibited a significantly lower plasma content of malondialdehyde (MDA) (*P<0.05*). The groups with 0.06% and 0.09% TB supplementation in feed exhibited significantly increased plasma activity levels of total superoxide dismutase (T-SOD) and glutathione peroxidase (GPx) (*P<0.05*). Furthermore, 0.03%-0.15% TB supplementation in feed significantly increased the catalase (CAT) activity in plasma (*P<0.05*). However, TB supplementation in feed had no significant effect on the plasma content of glutathione (GSH) or the activity of total antioxidant capacity (T-AOC) (*P>0.05*) (**Figures 1A, B**).

**Figure 2:**
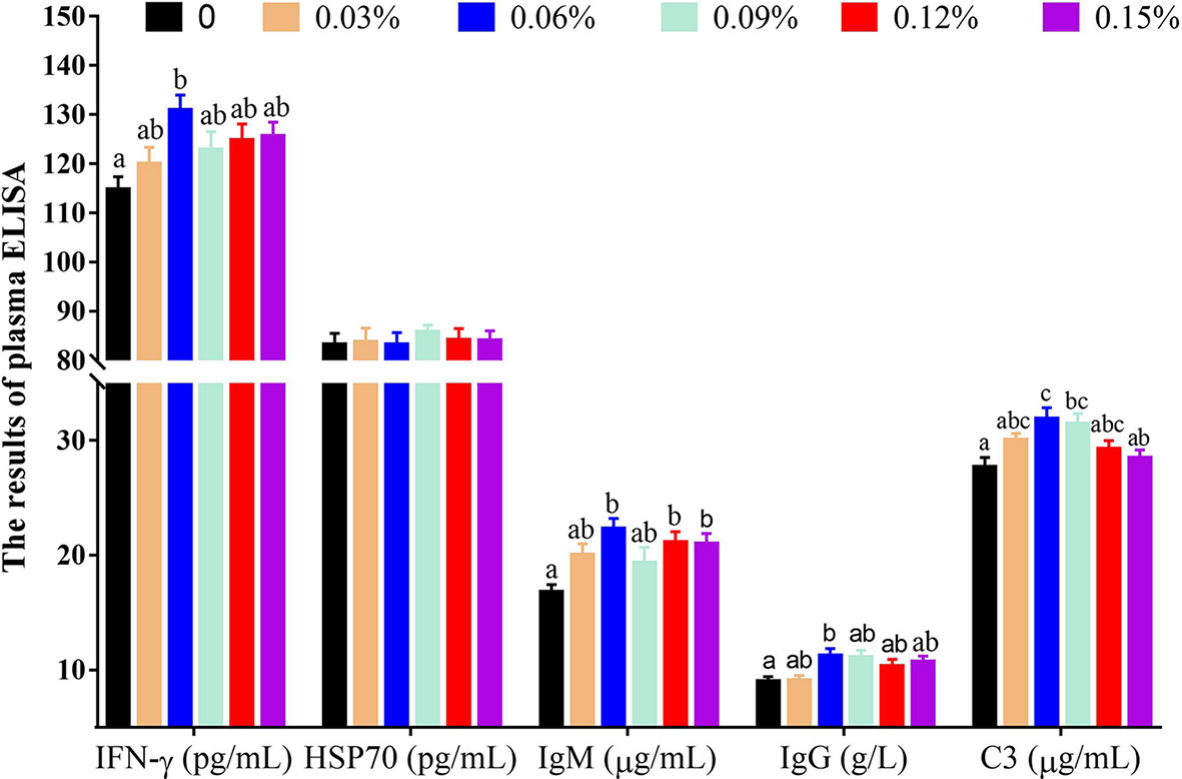
The contents of plasma immune factors with different levels of TB supplementation. IFN-γ, interferon-γ; HSP70, heat stress protein 70; IgM, immunoglobulin M; IgG, immunoglobulin G; C3, complement component 3. Data are expressed as means with S.E. Value with different superscripts are significantly different (P < 0.05).

**Figure 3:**
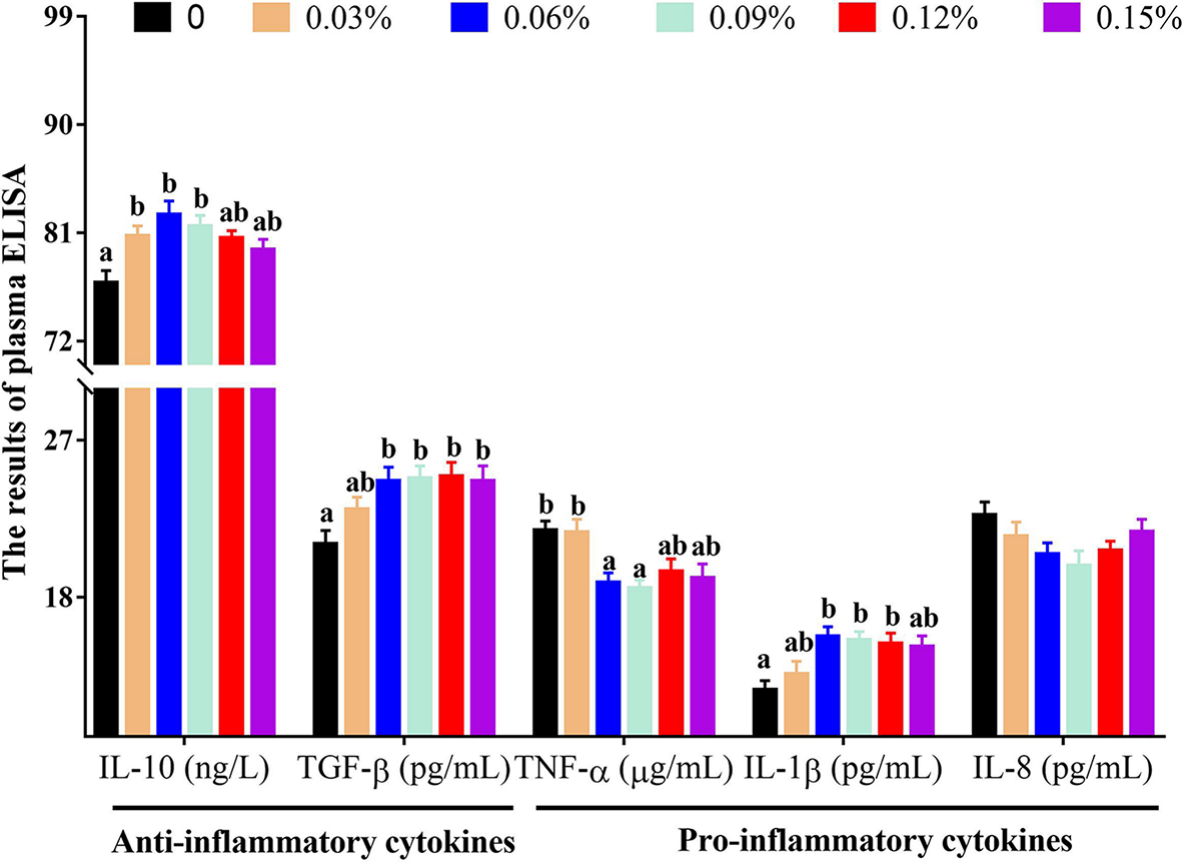
The contents of plasma inflammatory cytokines with different levels of TB supplementation. IL-10, interleukin 10; TGF-β, transforming growth factor-β; TNF-α, tumor necrosis factor-α; IL-1β, interleukin 1β; IL-8, interleukin 8. Data are expressed as means with S.E. Value with different superscripts are significantly different (P < 0.05).

Plasma ELISA revealed that compared with the control group (without TB supplementation, 0%), the group with 0.06% TB supplementation in feed exhibited significantly higher levels of IFN-γ, IgM and IgG (*P<0.05*), and the groups with 0.06% and 0.09% TB supplementation in feed exhibited significantly higher content of C3 (*P<0.05*) (**Figure 2**). With regard to inflammatory cytokines, compared with the control group, the groups with 0.03%-0.09% and 0.06%-0.15% TB supplementation in feed exhibited significantly higher levels of IL-10 and TGF-β (anti-inflammatory cytokines), respectively (*P<0.05*). The groups with 0.06% and 0.09% TB supplementation in feed exhibited significantly reduced levels of TNF-α (pro-inflammatory cytokine) (*P<0.05*) (**Figure 3**). However, TB supplementation in feed had no significant effects on the levels of HSP70 and IL-8 (*P>0.05*) (**Figures 2** and **3**). Surprisingly, 0.06%-0.12% TB supplementation significantly increased the content of IL-1β (*P<0.05*) (**Figure 3**).

### Relative Expression of Genes in the Intestine

**Figure 4:**
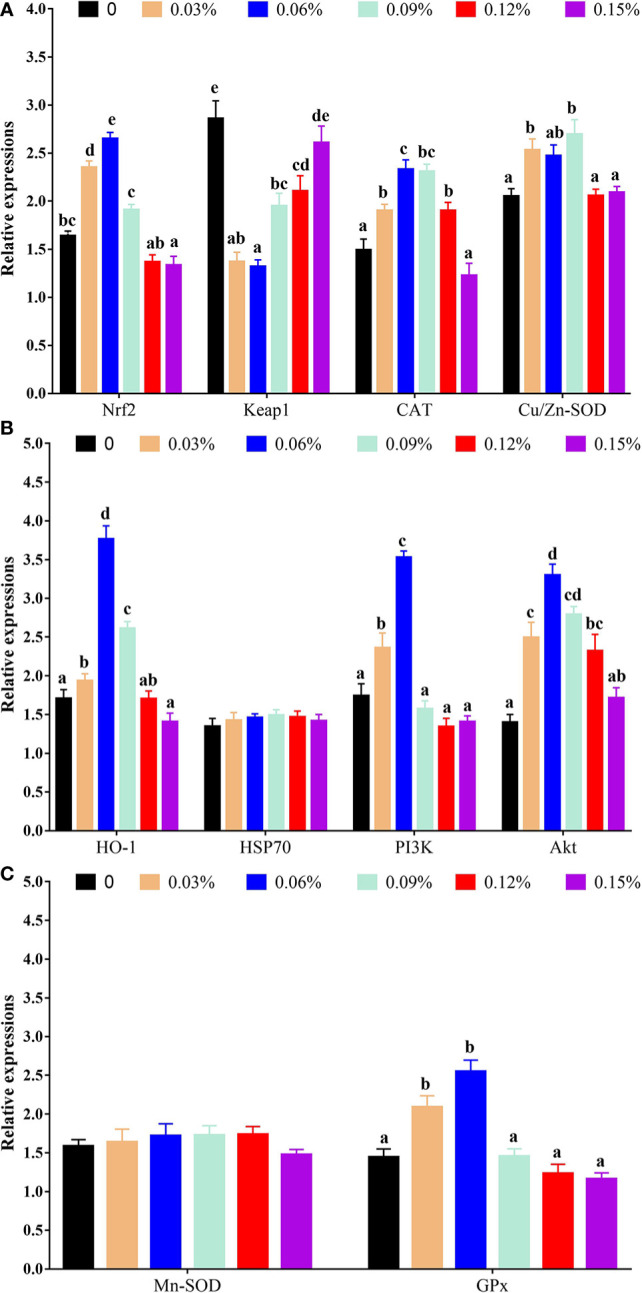
The relative expressions of vital gene in PI3K/Akt/Nrf2 signaling pathway with different levels of TB supplementation. **(A)** Nrf2, Nuclear factor erythroid 2-related factor 2; Keap1, Kelch-like ECH-associated protein 1; CAT, Catalase; Cu/Zn-SOD, Copper zinc superoxide dismutase; **(B)** HO-1, heme oxygenase 1; HSP70, heat stress protein 70; PI3K, phosphoinositide 3-kinase; Akt, protein kinase B; **(C)** Mn-SOD, manganese superoxide dismutase; GPx, glutathione peroxidase. Value with different superscripts are significantly different (P < 0.05).

**Figure 5:**
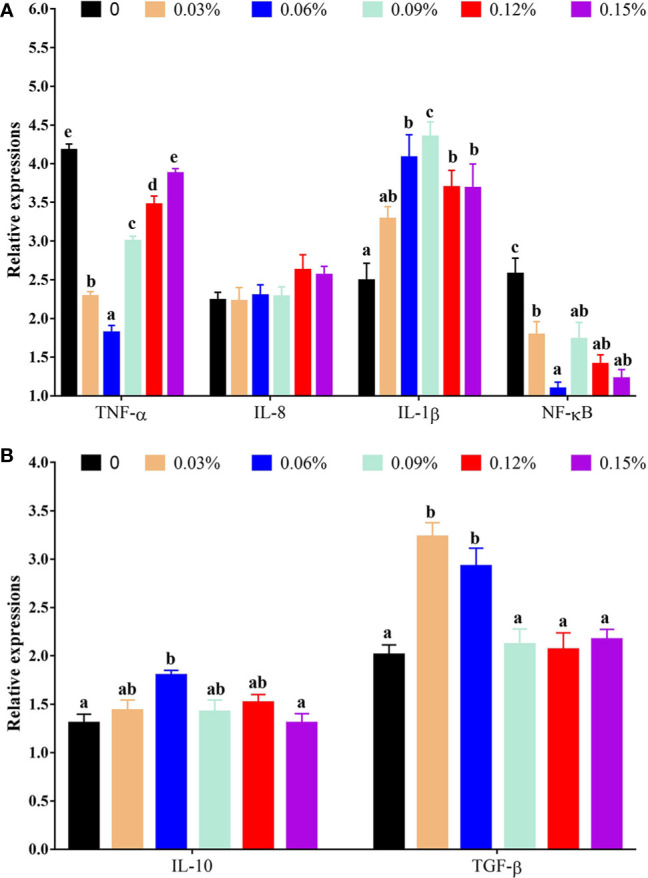
The relative expressions of vital gene in NF-κB signaling pathway with different levels of TB supplementation. **(A)** TNF-α, tumor necrosis factor-α; IL-8, interleukin 8; IL-1β, interleukin 1β; NF-κB, nuclear factor-kappa **(B)**; **(B)** IL-10, interleukin 10; TGF-β, transforming growth factor-β. Data are expressed as means with S.E. Value with different superscripts are significantly different (P < 0.05).

With regard to antioxidant genes, the relative expression of nuclear factor erythroid 2-related factor 2 (Nrf2) showed a trend of increasing first and then decreasing with the addition levels of TB in feed, and the highest levels was observed in 0.06% TB supplementation in feed (*P<0.05*), however, the relative expression of Kelch-like ECH-associated protein 1 (Keap1) showed an opposite trend with Nrf2. Furthermore, with the addition levels of TB in feed, the relative expression of CAT, protein kinase B (Akt), heme oxygenase 1 (HO-1), phosphoinositide 3-kinase (PI3K) and GPx were increased with increasing dietary TB supplementation level up to 0.06% (*P<0.05*), and thereafter showed a decreasing trend, while, the highest expression levels of copper and zinc superoxide dismutase (Cu/Zn-SOD) was observed in 0.09% TB supplementation in feed (*P<0.05*) (**Figure 4**). With regard to inflammatory cytokines, 0.03%-0.15% TB supplementation in feed significantly reduced the relative expression of nuclear factor-kappa B (NF-κB) and 0.03%-0.12% TB supplementation in feed significantly reduced the relative expression of TNF-α. Furthermore, 0.06% and 0.03%-0.06% TB supplementation in feed significantly increased the relative expression of IL-10 and TGF-β, respectively (*P<0.05*). Surprisingly, 0.06%-0.15% TB supplementation significantly increased the mRNA levels of IL-1β (*P<0.05*). However, TB supplementation in feed had no significant effect on the relative expression of HSP70, manganese superoxide dismutase (Mn-SOD) or IL-8 (*P>0.05*) (**Figures 4** and **5**).

### *Aeromonas hydrophila* Challenge Test

**Figure 6:**
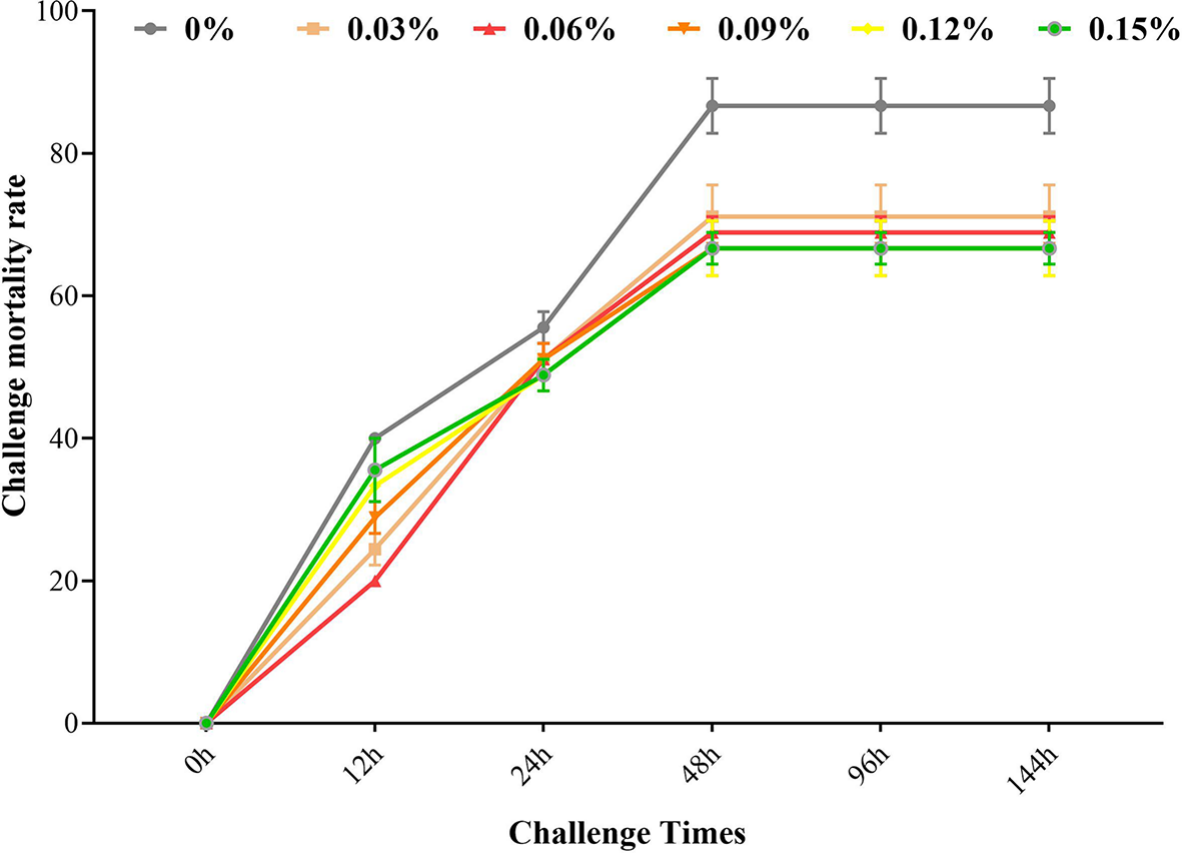
Mortality rate with *Aeromonas hydrophila* challenge after during 144h with different levels of TB supplementation. Data are expressed as means with S.E.

After *A. hydrophila* challenge, the highest mortality rate of blunt snout bream was observed among fish given food with 0% TB supplementation at 144 h (*P<0.05*). The lowest mortality rate of blunt snout bream was observed among fish given food with 0.09% and 0.12% TB supplementation at 144 h (*P<0.05*) (**Figure 6**).

### Hepatic and Intestinal Histopathology

**Figure 7:**
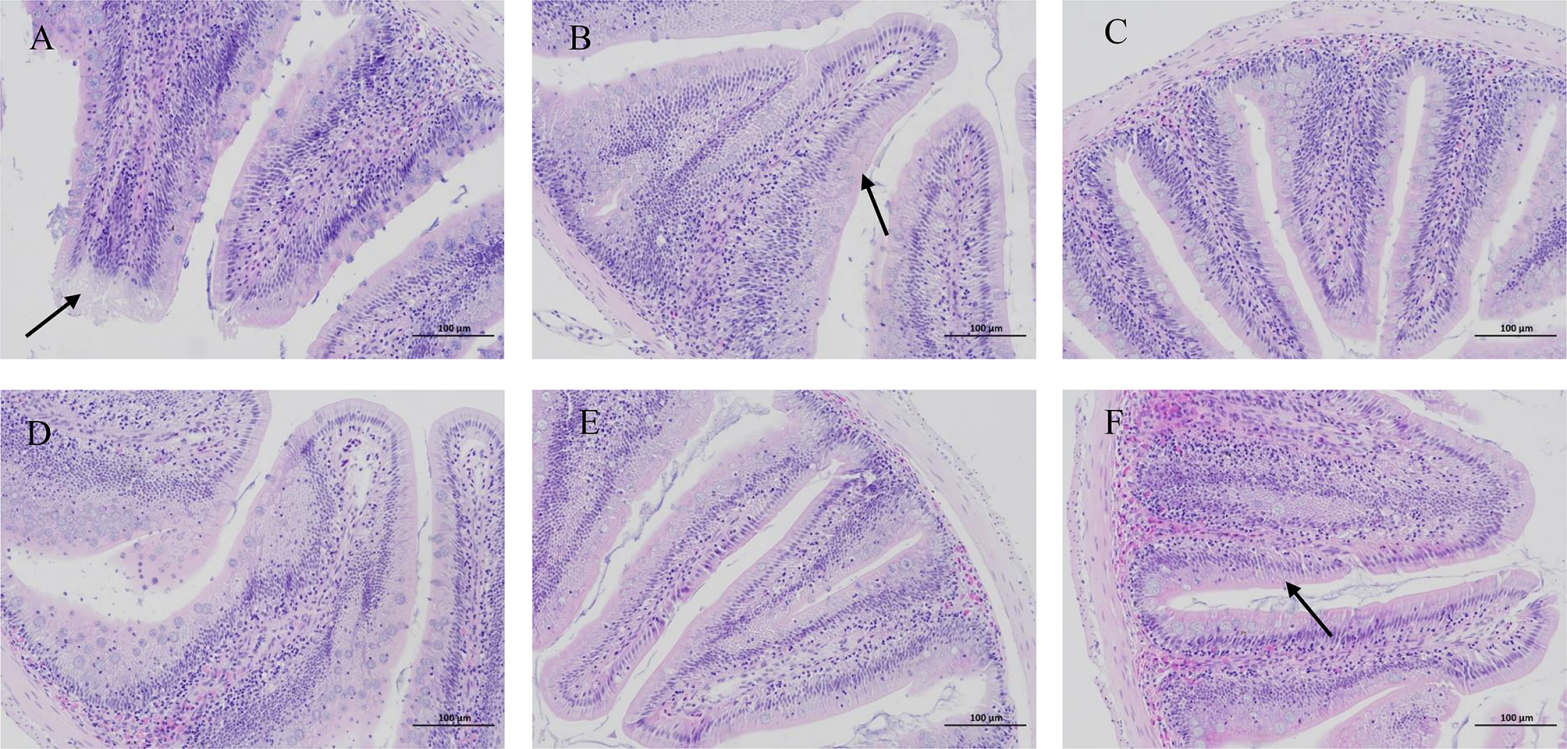
The intestinal HE staining of juvenile blunt snout bream (200X). **(A–F)** were corresponding to 0%, 0.03%, 0.06%, 0.09%, 0.12% and 0.15% TB supplementation. The black arrow indicated that there was lysis and necrosis at the top of the intestinal villi, and the cell structure disappeared **(A)**; a small number of intestinal villi fused with each other and the intestinal villi become wider **(B, F)**.

**Figure 8:**
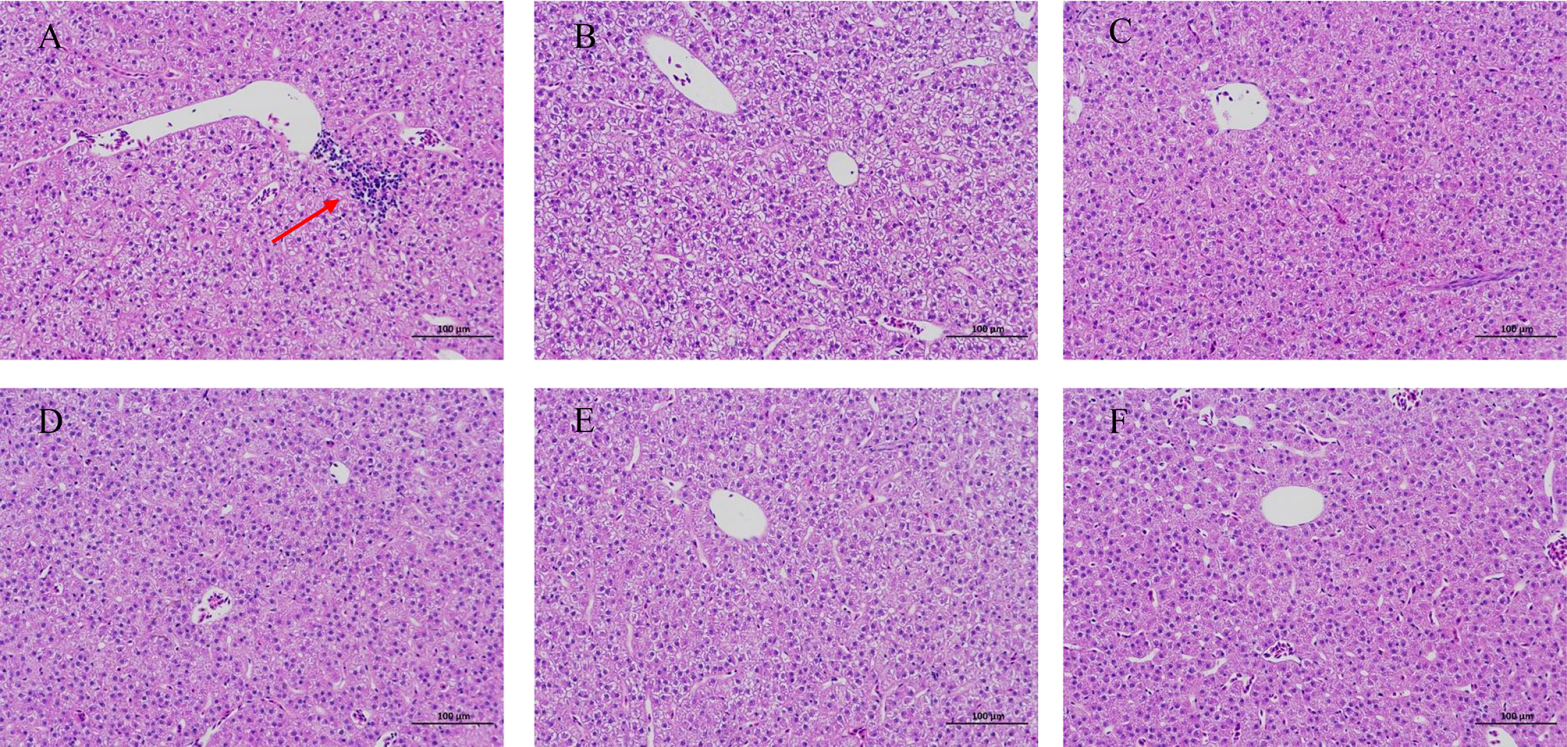
The hepatic HE staining of juvenile blunt snout bream (200X). **(A–F)** were corresponding to 0%, 0.03%, 0.06%, 0.09%, 0.12% and 0.15% TB supplementation, respectively. The red arrow indicated that a small number of inflammatory cells were locally infiltrated **(A)**.

HE staining of juvenile blunt snout bream intestinal tissue revealed lysis and necrosis at the tips of the intestinal villi and indicated that the cell structure disappeared in fish given food with 0% TB supplementation. Furthermore, in the groups of fish given diet with 0.03% and 0.15% TB supplementation, a small number of intestinal villi fused with each other, and the intestinal villi became wider; however, in other groups, the structure of each layer of the intestine was clear, the mucosal epithelial cells were not shed, the intestinal villi were abundant and arranged regularly, and goblet cells were visible (**Figure 7**). Hepatic HE staining of juvenile blunt snout bream tissues revealed that a small number of inflammatory cells were locally infiltrated in fish given food with 0% TB supplementation; however, tissues from fish in other groups, the hepatic cells were arranged neatly with clear outlines, and the hepatic sinusoids were normal (**Figure 8**).

## Discussion

### Effect of TB Supplementation on Growth and Whole-Body Composition

TB has better palatability than butyric acid or sodium butyrate because it has almost no smell or only a slightly fatty fragrance, and it has great potential for use in aquatic feed. In this study, the growth performance results showed that TB supplementation in feed had some positive impacts on FW, WG, FCR and SGR, and the best results were found in the 0.06% TB group. These results indicated that TB supplementation in feed was effective in enhancing fish performance, at least in *Megalobrama amblycephala*. Researchers have demonstrated the availability and functionality of TB supplemented in the feed of terrestrial animals, such as broiler chickens (9, 10), weaned pigs (11), and small tail sheep ewes (12). Among aquatic animals, the effects of TB supplementation in feed have also been assessed in some fish species. TB supplementation in feed can also improve the growth performance of snakehead (18), black sea bream (19) and juvenile yellow drum (20, 21). The relevant mechanism may be that TB is decomposed into butyric acid under the action of intestinal lipase, promoting the proliferation and differentiation of mucosal cells after absorption by the intestine (31), which can expand the digestion and absorption area to improve intestinal physical barrier function, nutrient absorption, and nutrient utilization (20, 31). In addition, TB supplementation in feed had no significant effect on CF, VSI or whole-body composition of components including protein, lipids, moisture and ash, similar to the case in black sea bream (19), however, Tan et al. (20) reported that TB supplementation in a high soya bean meal diet significantly reduces body fat content in juvenile yellow drum. These differing results may have occurred because of the different species, different amounts of added butyrate, different durations of the experiments, and other differences.

### Effect of TB Supplementation on Antioxidant Status

Antioxidant status plays a vital role in the growth of fish (27, 30). In the present study, compared with the control group (0% TB supplementation), the group with 0.06% TB supplementation in feed improved antioxidant capacity, as indicated by reduced plasma levels of MDA and increased plasma activity of T-SOD and GPx, suggesting that TB can alleviate or reduce intestinal oxidative stress. TB supplementation effectively decreases MDA content in piglets challenged with diquat (32). Jiang et al. (33) discovered that sodium butyrate reduces MDA levels in broiler chickens challenged with corticosterone. TB supplementation (0.05%) significantly improves intestinal antioxidative capacity in LPS-challenged broilers (34). Among aquatic animals, TB supplementation effectively improves oxidative capacity in snakehead (18) and black sea bream (19). The activity levels of enzymes affect the corresponding gene expression (25, 27). Studies have shown that the PI3K/Akt signaling pathway is crucial for cell survival, inhibition of the development of cancer cells and the antioxidative system (25, 35). In this study, 0.03% and 0.06% TB supplementation in feed significantly improved PI3K and Akt mRNA levels. Furthermore, various reports have demonstrated that the Nrf2-Keap1 signaling pathway plays an important role in regulating antioxidative capacity (36, 37). In this study, 0.03% -0.06% or 0.09% TB supplementation in feed significantly increased the relative expression of Nrf2, Cu/Zn-SOD, HO-1, CAT, and GPx and reduced the relative expression of Keap1, which was correlated with PI3K and Akt mRNA levels. There were evidences that Nrf2 signaling was activated *via* the PI3K/Akt pathway (38, 39). In our previous studies, we also found that Nrf2 signaling pathway has a Pearson correlation with the PI3K/Akt pathway and Nrf2 signaling was activated *via* the PI3K/Akt pathway in blunt snout bream (25, 40). The results of this study suggest that the optimum TB supplementation may induce Nrf2/Keap1 pathway signaling partly by activating the PI3K/Akt pathway, which further regulates antioxidant gene expression and the activity of related enzymes to improve the antioxidant capacity of blunt snout bream. The possible mechanism is that TB supplementation can increase the content of adenosine triphosphate (ATP) (34), which plays an important role in activating PI3K/Akt signaling (41), further activating the Nrf2 signaling pathway to regulate antioxidant ability.

### Effect of TB Supplementation on Immunocompetence

Immunity is an important factor in the maintenance of healthy growth and disease resistance in animals (42). Immunoglobulins, the complement system and interferons play important roles in immune regulation in humans and animals (43–45). Hence, we also investigated the effect of TB on immunity. In the present study, 0.06% TB supplementation in feed improved immunocompetence by increasing the levels of IFN-γ, IgM, IgG and C3 in plasma. Similarly, various previous studies have reported that TB or butyric acid supplementation in feed can also increase the production of immunoglobulins (13) and IFN-γ (44). Furthermore, IL-10 and TGF-β are two important anti-inflammatory cytokines (16, 46), and TNF-α and IL-8 are two important pro-inflammatory cytokines (46). In the present study, 0.06% and 0.09% TB supplementation in feed significantly increased the levels of IL-10 and TGF-β and significantly decreased the levels of TNF-α. TB supplementation significantly decreases the levels of TNF-α in the plasma of rats after LPS administration (47). Studies have reported that TB supplementation can increase the levels of IL-10 in retroperitoneal adipose tissue (46) and colitis+TBT mice (16), and the content of TGF-β is also increased by TB supplementation in experimental colitis (16). These results are consistent with our results and indicate that TB supplementation can improve immunocompetence by regulating the production of inflammatory cytokines. Interestingly, in mice fed with tributyrin-supplemented diet, the levels of TNF-α have been found to be elevated principally in retroperitoneal and epididymal adipose tissue (46). These differing results may have occurred because of the different amounts of supplemented TB, the different durations of the experiments or the different study species.

TB supplementation attenuates the inflammatory response *via* inhibition of NF-kB activation (47, 48). In the present study, 0.03%-0.15% TB supplementation inhibited the relative expression of NF-κB, and the lowest expression was observed in fish fed the diet with 0.06% TB supplementation. Based on these results, TB supplementation might have a positive effect on the inflammatory response in this fish. Studies have demonstrated that NF-κB is a redox-responsive transcription factor that regulates the expression of downstream inflammatory cytokines, including pro-inflammatory cytokines and anti-inflammatory cytokines (49). In the present study, the 0.06% TB supplementation group showed the lowest expression of TNF-α and the highest expression of IL-10, while the 0.03% TB supplementation group showed the highest expression of TGF-β, which were positively correlated or negatively correlated with NF-κB expression. Various studies have reported that TB or butyrate supplementation can increase the mRNA levels of anti-inflammatory cytokines, including IL-10 in the distal intestines of juvenile yellow drum (21) and TGF-β in intestinal epithelial-like Caco-2 cells (50), and can reduce the mRNA levels of the pro-inflammatory cytokine TNF-α in piglets challenged with diquat (32), rats challenged with LPS (47) and juvenile yellow drum fed a SO-based diet with 0.20% TB (21). These results support the conclusion that TB supplementation attenuates the inflammatory response *via* inhibition of NF-kB activation to regulate pro-inflammatory cytokines and anti-inflammatory cytokines. The possible mechanism is that TB might activate PI3K/Akt, which further regulates the NF-κB signaling pathway (51–53). In the *A. hydrophila* challenge test, a lower mortality rate was observed in the groups of blunt snout bream fed diets with 0.06%-0.15% TB supplementation at 144 h than in the other groups, which also supports the results for the immune indexes and antioxidant indexes in this study. Interestingly, the mRNA and content of the pro-inflammatory cytokine IL-1β were increased by TB supplementation in this study, similar to the effects of TB supplementation in experimental colitis (16). This may be because cytokines play other roles in immune regulation. IL-1β might play major roles in the improvement of mucosal architecture, cell proliferation, and mucosal regeneration, rather than a role of pro-inflammatory cytokine (16). Furthermore, other studies have also reported that IL-1β might be involved in regulating several important physiological processes, including cell proliferation, differentiation and apoptosis (54, 55). In contrast to these results, Li et al. (34) reported that TB supplementation decreases the levels of pro-inflammatory cytokines such as IL-1β in the intestines of broilers challenged with LPS. Hence, the relevant mechanisms need to be further explored.

### Protective Effects of TB Supplementation on the Liver and Intestine

In the present study, appropriate TB supplementation had positive protective effects on the liver and intestine, preventing lysis and necrosis of the intestine and inflammatory cell infiltration of the liver. TB supplementation or oral administration exerts important restorative effects in the liver or intestines, such as in growth‐retarded piglets (13), lipopolysaccharide-treated rats (47), snakehead (18), black sea bream (19) and yellow drum (20). These findings are in agreement with the results of the present study. The possible reason is that TB can activate the Nrf2 signaling pathway and inhibit the NF-κB signaling pathway, which further improves antioxidant capacity and attenuates the inflammatory response. Other possible reasons are that butyrate ions can regulate the intestinal mucosa and repair damage to the intestinal mucosa; TB can not only release sufficient butyrate ions at designated points in the intestine but also provide necessary energy to complete the repairs, and the liver is able to take up butyrate in patients with normal liver function (56).

## Conclusion

**Figure 9:**
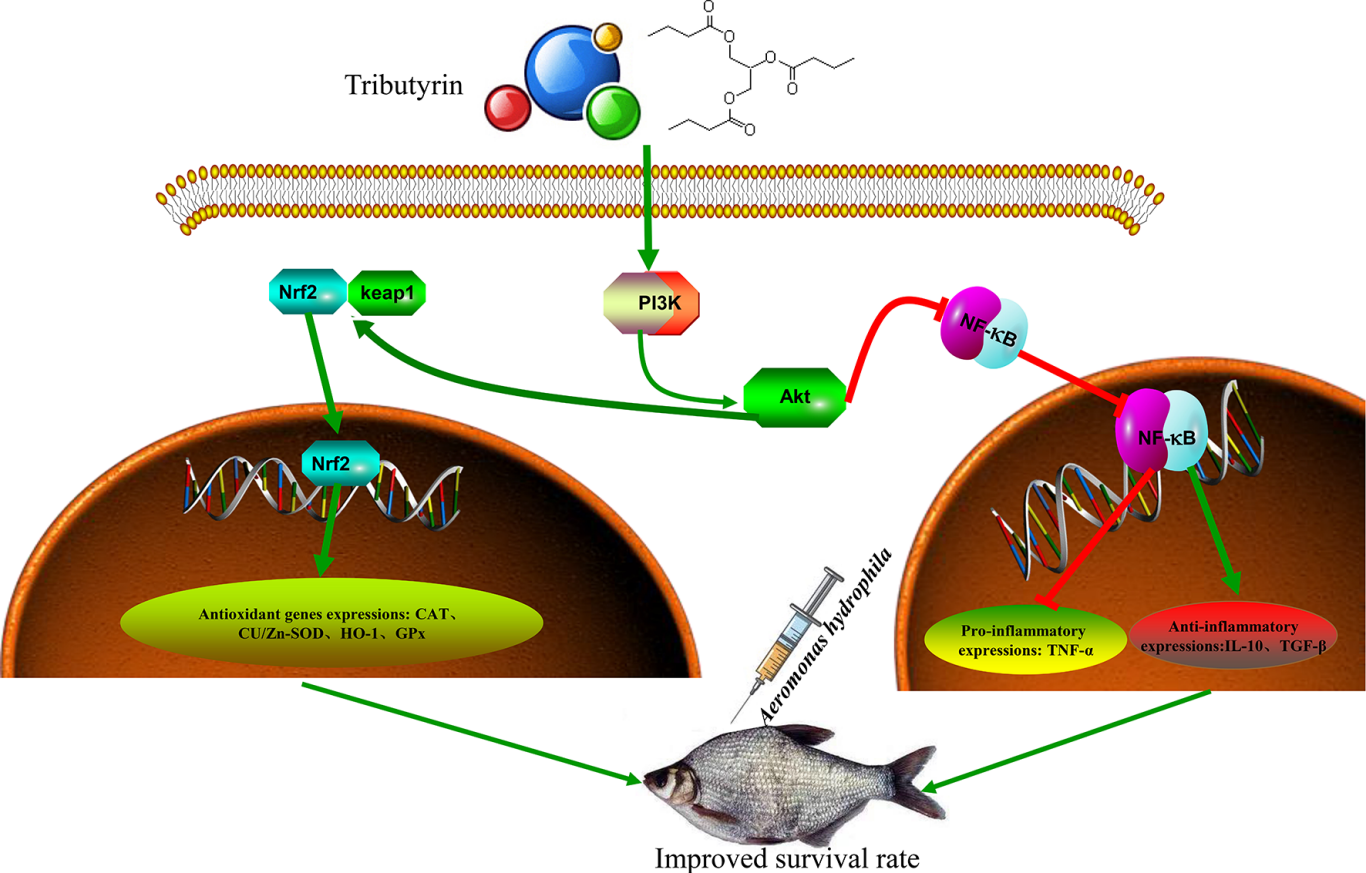
Regulation Mechanism of improving health status by tributyrin in *Megalobrama amblycephala*.

TB supplementation improves the growth performance of juvenile blunt snout bream. Furthermore, TB supplementation activates PI3K/Akt/Nrf2 and inhibits the NF-κB signaling pathway, regulating antioxidant capacity and the inflammatory response and preventing hepatic and intestinal damage (**Figure 9**). Based on the growth performance and immunocompetence, the recommended additive amount was 0.06% in the feed of juvenile blunt snout bream.

## Data Availability Statement

The raw data supporting the conclusions of this article will be made available by the authors, without undue reservation.

## Ethics Statement

The animal study was reviewed and approved by The protocols used on the experimental fish followed the guidelines of the Institutional Animal Care and Ethics Committee of Nanjing Agricultural University, Nanjing, China. [Permit number: SYXK (Su) 2011-0036].

## Author Contributions

MR and LZ designed the study. HL carried out the experiments and wrote the manuscript. KJ provided technical assistance. BX provided technical guidance. XC provided technical guidance. All authors contributed to the article and approved the submitted version.

## Funding

This study was financially supported by the National Key R&D Program of China (2019YFD0900200), the Natural Science Foundation of Jiangsu Province (BK20200169), National Natural Science Foundation of China, NSFC (31772820), the Modern Agriculture Industrial Technology System special project the National Technology System for Conventional Freshwater Fish Industries (CARS-45).

## Conflict of Interest

LZ and XC were employed by company Tongwei Co., Ltd.

The remaining authors declare that the research was conducted in the absence of any commercial or financial relationships that could be construed as a potential conflict of interest.
